# *Anopheles mascarensis*, a Malaria Vector Endemic to Madagascar and the Comoros Archipelago: A Review

**DOI:** 10.4269/ajtmh.24-0697

**Published:** 2025-10-28

**Authors:** Jessy Goupeyou-Youmsi, Luciano M. Tantely, Tsarasoa M. Andrianinarivomanana, Romain Girod, Catherine Bourgouin

**Affiliations:** ^1^ Immunology of Infectious Diseases Unit, Institut Pasteur de Madagascar, Antananarivo, Madagascar;; ^2^ Functional Genetics of Infectious Diseases, Institut Pasteur, Université de Paris Cité, Paris, France;; ^3^ Doctoral School Complexité du Vivant, Sorbonne Université, Paris, France;; ^4^ Medical Entomology Unit, Institut Pasteur de Madagascar, Antananarivo, Madagascar;; ^5^ UMR2000-CNRS, Institut Pasteur, Paris, France

## Abstract

*Anopheles mascarensis* (*An. mascarensis*; De Meillon, 1947) is a mosquito species endemic to Madagascar and the Comoros Archipelago. In the past, it was confused with *Anopheles marshalli* (Theobald, 1929), a continental African species that does not exist in Madagascar. Malaria transmission is highly heterogeneous in Madagascar. Principal and secondary mosquito vectors, as well as malaria parasite species, may vary from one region to another. *Anopheles mascarensis* has been identified as the main vector of malaria in the east and southeast of Madagascar, while it plays the role of a secondary vector in other Malagasy regions. Differences in behavior between *An. mascarensis* populations from the east coast and those from the Central Highlands of Madagascar suggest that *An. mascarensis* may be composed of sibling species. In the present review, unpublished data on the geographical distribution of *An. mascarensis* were assembled to update the previous distribution map published in 1966. In addition, published data on the biology of this mosquito, its geographical variants, and records of its role in malaria transmission were analyzed. The published data highlight a significant difference between populations from the east coast and those from the Central Highlands, revealing a possible gradient along different climatic and biogeographic regions of Madagascar. This analysis supports the idea that *An. mascarensis* may consist of a complex of sibling species. With advances in molecular tools, testing this hypothesis is increasingly within reach.

## INTRODUCTION

*Anopheles mascarensis* (*An. mascarensis*) is a mosquito species endemic to Madagascar and the Comoros Archipelago. It was described by De Meillon in 1947^1^ on the basis of unique adult samples collected in Diego Suarez (which is actually Antsiranana), at the northern tip of Madagascar. It was named after the Mascarene Islands, which include Mauritius, La Réunion, and Rodrigues, located on the eastern side of Madagascar.^2^ It has long been confused with *Anopheles marshalli* (*An. marshalli*) Theobald, a continental African species that does not exist in Madagascar.^3^^,^^4^ *Anopheles mascarensis* belongs to the *Cellia* subgenus of the *Anopheles* genus, which includes many important malaria vectors in Africa and Asia.^5^^,^^6^ Several entomologists have reported subtle but significant morphological variations suggesting the existence of geographical variants of *An. mascarensis*.^3^ During numerous malaria surveillance campaigns over the last century, the presence of *An. mascarensis* was reported in several places in Madagascar, but it was never implicated as a potential malaria vector until 1992.^3^^,^^7^^,^^8^ In 1992, Fontenille and colleagues^9^^,^^10^ found that *An. mascarensis* was an important secondary vector of human malaria on Sainte Marie Island, located off the east coast of Madagascar. In 1999, Marrama et al. reported that *An. mascarensis* acted as the primary malaria vector in a locality near Fort Dauphin (which is actually Tolagnaro) in the tropical southeastern region of Madagascar.^11^

**Figure 1.**
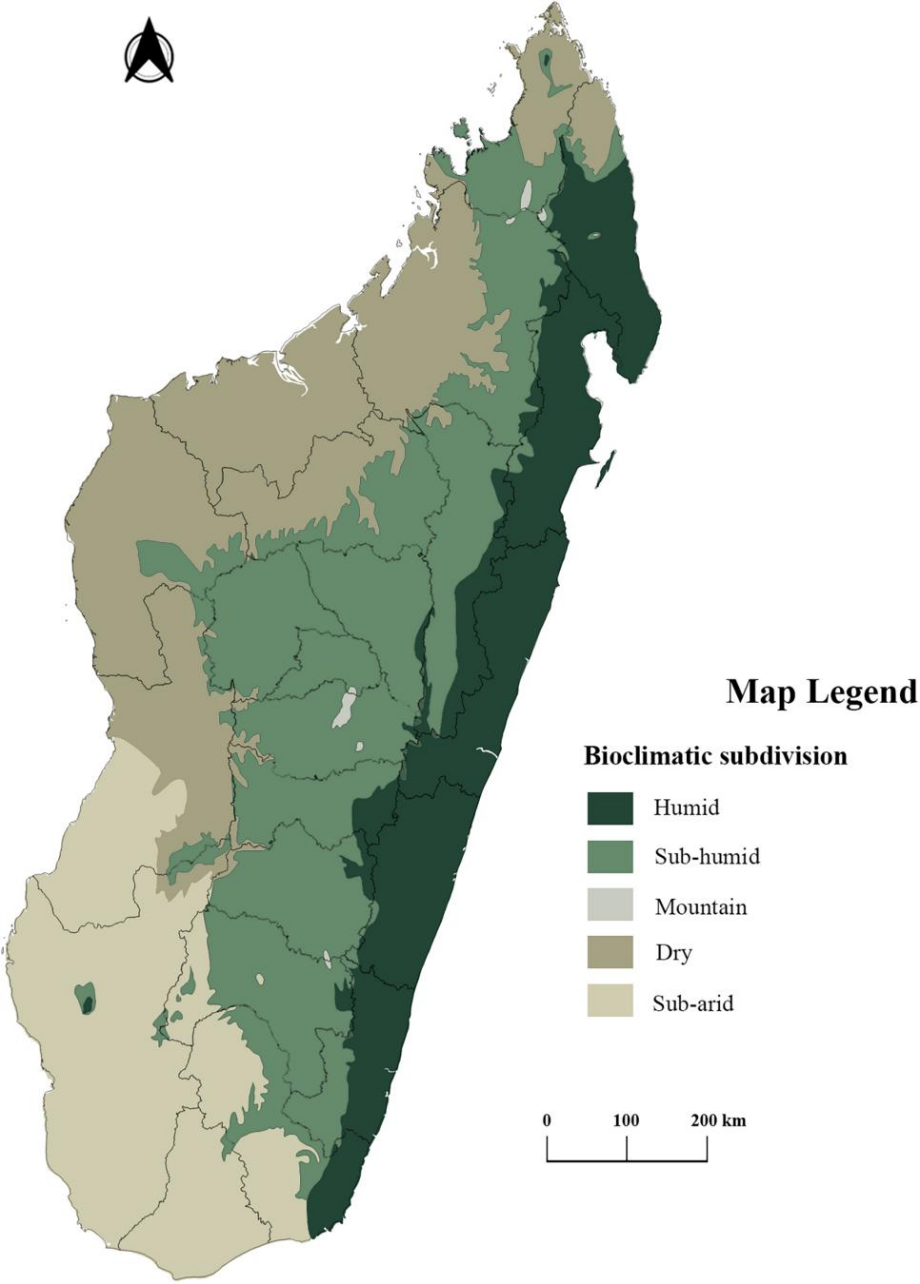
Madagascar’s bioclimatic domains. Adapted from Cornet 1973 (https://datasuds-geo.ird.fr/geonetwork/srv/resources/records/0839b2e5-596a-48e5-8678-1af779dffa72), with modifications performed by the Geomatic Group of the Epidemiology Unit at the Institut Pasteur de Madagascar.

The epidemiology of malaria transmission in Madagascar is complex because of the geography of the country and its climatic stratification.^12^^–^^15^ A topographical map of Madagascar can be found at https://commons.wikimedia.org/wiki/File:Madagascar_Topography.png. Five bioclimatic domains have been proposed by Cornet^16^ (Figure 1). A brief description of these bioclimatic domains can be found in Pock Tsy et al.^17^ *Anopheles funestus* (*An. funestus*), *Anopheles gambiae* s.s. (*An. gambiae*), and *Anopheles arabiensis* are the main malaria vectors in most regions of Madagascar. The tremendous malaria vector control efforts put in place after the devastating malaria epidemics that occurred between 1986 and 1988 revealed changes in malaria vector distribution, notably the reemergence of *An. funestus* as a major malaria vector in the Central Highlands.^18^^–^^21^ Despite continuous malaria control efforts, malaria remains a major health problem in Madagascar, which is currently facing both climatic and societal changes that have increased poverty in areas where malaria has reestablished itself as a major threat.^22^^,^^23^ The situation has worsened after the coronavirus disease 2019 pandemic.^24^ In this context, whether *An. mascarensis* acts as a locally secondary or major vector remains an open question. Furthermore, the descriptions of geographical, morphological, and behavioral variants over the last 50 years suggest that *An. mascarensis* may comprise a complex of sibling species with possibly different vectorial capacities.^3^^,^^9^

**Figure 2.**
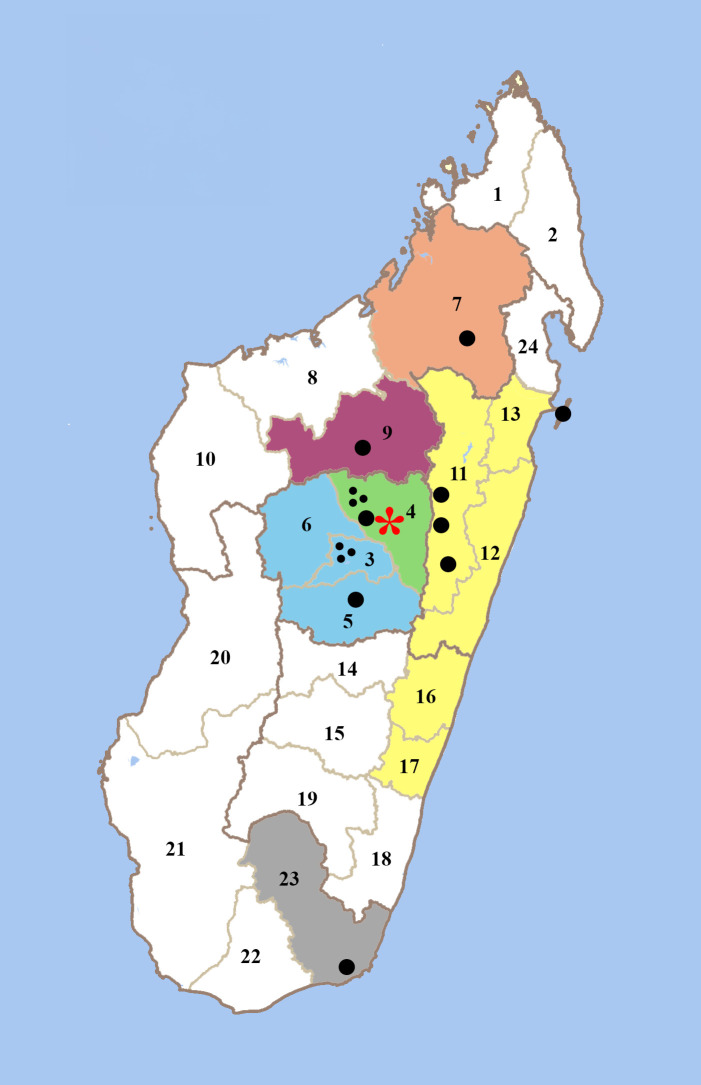
Localization of the field sites where the presence of *Anopheles mascarensis* was reported (1990–2024). The limits of the 24 different administrative regions are indicated, as well as their official numbered code according to https://fr.wikipedia.org/wiki/R%C3%A9gions_de_Madagascar#/. Black dots = sampled sites; blue = western fringe; gray = tropical southeast; green = Central Highlands; orange = northeast; purple = northwestern fringe; red star = Antananarivo; yellow = east coast.

In the present review, published data on the geographical distribution of *An. mascarensis*, its biology, and its contribution to malaria transmission were analyzed. The list of publications used is presented in Table 1, and the localization of the investigated sites is shown in Figure 2. Furthermore, information was extracted from the database of the Medical Entomology Unit at the Institut Pasteur de Madagascar, spanning the period from 1989 to 2015. The current work provides a foundation for further investigation into whether *An. mascarensis* is indeed a complex of species with distinct geographical distributions, behaviors, and malaria vectorial capacities.

**Table 1:** List of publications used to create Figures 2 and 5

Authors	Date of Publication	Link
Fontenille, Lepers, et al.	1992	https://www.ncbi.nlm.nih.gov/pubmed/1495029
Andrianaivolambo, Domarle, et al.	2010	http://www.sciencedirect.com/science/article/pii/S0001706X10002287
Tantely, Rakotoniaina, et al.	2013	https://www.ncbi.nlm.nih.gov/pubmed/23802456
Marrama, Laventure, et al.	1999	https://www.ncbi.nlm.nih.gov/pubmed/10399606
Ravoahangimalala, Rakotoarivony, et al.	2003	https://www.ncbi.nlm.nih.gov/pubmed/14717053
Rajaonarivelo, Le Goff, et al.	2004	https://doi.org/10.1051/parasite/200411175
Le Goff, Randimby, et al.	2003	https://www.ncbi.nlm.nih.gov/pubmed/15678818
Laganier, Randimby, et al.	2003	https://doi.org/10.1186/1475-2875-2-42
Robert, Le Goff, et al.	2006	http://www.sciencedirect.com/science/article/pii/S0020751906001895
Fontenille, Lepers, et al.	1990	https://www.ajtmh.org/view/journals/tpmd/43/2/article-p107.xml
Nepomichene, Tata, et al.	2015	https://www.ncbi.nlm.nih.gov/pubmed/26620552
Goupeyou-Youmsi, Rakotondranaivo, et al.	2020	https://doi.org/10.1186/s13071-020-04282-0
Andrianinarivomanana Tsarasoa Malala	2024	https://www.researchgate.net/publication/393898815_Contribution_d'Anopheles_coustani_dans_la_transmission_des_parasites_du_paludisme_a_Madagascar_role_vecteur_et_diversite_genetique

The order follows the description according to each sampled region: east, tropical southeast, northeast, western fringe, Central Highlands, and northwestern fringe.

## RESULTS

### Systematic position.

According to Alexis Grjebine, *An. mascarensis* was once possibly named *Anopheles funesta* var *neireti* by Blanchard in 1906 and later *An. funestus* var *imerensis* by Monier and Treillard in 1935, on the basis of abundant samples collected around Antananarivo.^8^^,^^25^ Unfortunately, none of these mosquitoes were preserved. Until the holotype description by De Meillon in 1947, *An. mascarensis* was long described as *An. marshalli.*^1^ Even later, it was still often confused with this species, despite the typical morphological characteristics of the cibarial armature of the adults described by De Meillon.^3^^,^^4^ The larval and pupal stages were only described in 1961.^26^ In this latter publication, the authors discussed the presence or absence of some characteristics of *An. mascarensis* adults that could call into question the classification of this species within the Neomyzomyia series, as proposed by De Meillon. The most recent classification has placed *An. mascarensis* within the subgenous *Cellia* Theobald and the Neomyzomyia series.^27^ Within this series, a Mascarensis group that contains the sole *An. mascarensis* species was proposed.^28^ In contrast, *An. marshalli* Theobald belongs to the Myzomyia series of the *Cellia* subgenus. It was later classified in the Marshalli complex^29^ within the Marshalli group.^30^ This latter information was assembled by Harbach.^31^^,^^32^ Additional information can be found in Irish et al.^33^

### Geographical distribution.

**Figure 3.**
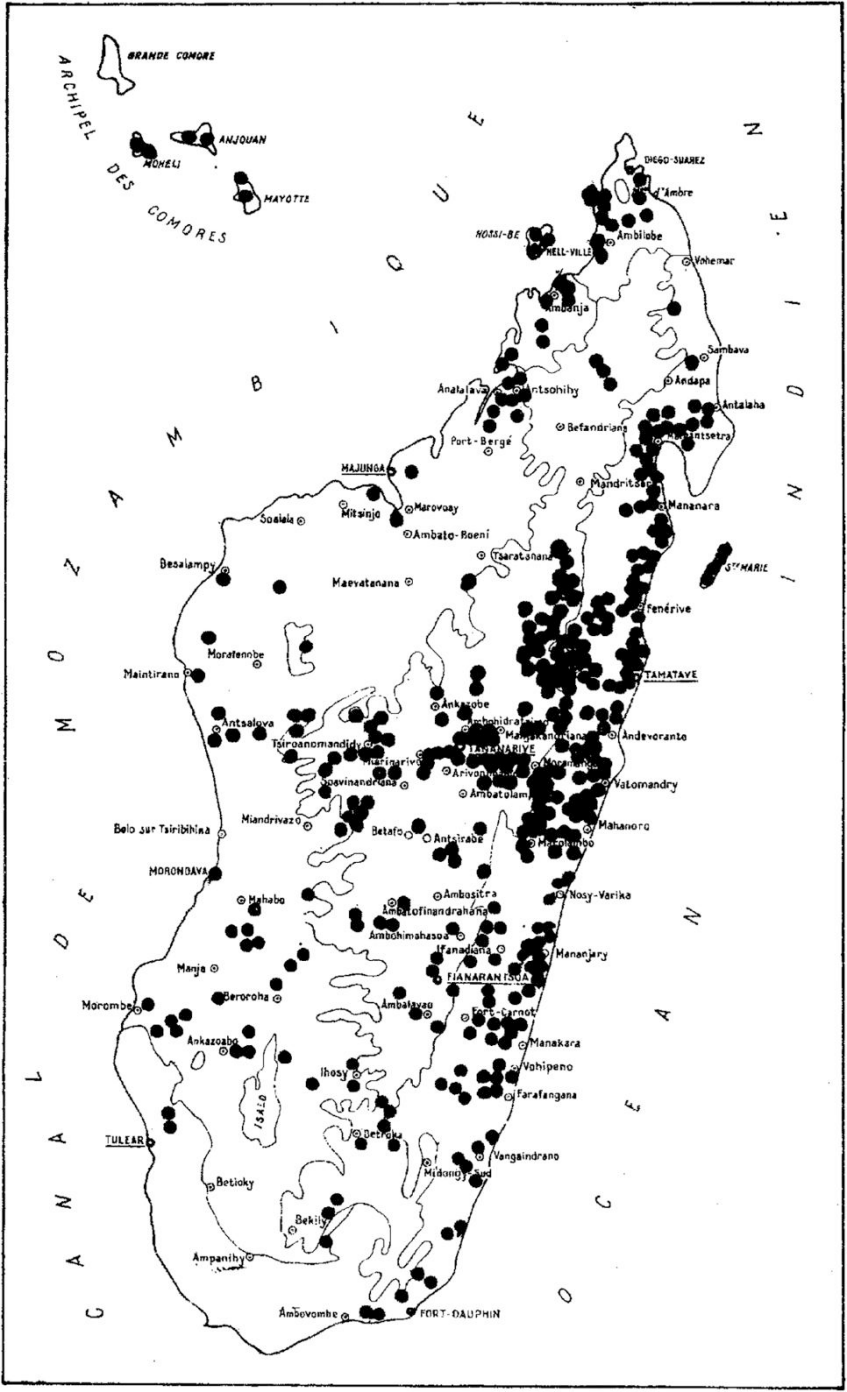
Geographical distribution of *Anopheles mascarensis* (Grjebine, 1966). Reproduction authorized by IRD Editions.

In his comprehensive review on Malagasy anophelines published in 1966, Grjebine assembled a wealth of information on both the chorology and biology of *An. mascarensis*, and provided the first distribution map of *An. mascarensis* in Madagascar and the Comoros Archipelago.^8^ Most records were established over a 10-year period from 1952 to 1962, revealing the presence of *An. mascarensis* in ∼400 localities across Madagascar (Figure 3). With the exception of the most southern arid part of Madagascar, *An. mascarensis* was widespread in every region and at all elevations, from 1,200 m to the coastline. It was also found in Anjouan, Moheli, and Mayotte in the Comoros Archipelago. Its abundance varied from one locality to another, with a trend toward higher abundance on the east coast of Madagascar (Ivoloina, Toamasina, Sainte-Marie Island, and Mananara).^3^^,^^7^^,^^8^^,^^10^^,^^26^ On the west coast, it was scarce in some localities.^7^^,^^8^^,^^34^ In the Central Highlands, it was found in many places from north to south and from the eastern to western fringes, as well as in the vicinity of Antananarivo, the capital city of Madagascar.

Published records on the occurrence of *An. mascarensis* are almost nonexistent from 1966 to 1988. However, during arbovirus prevalence surveys in the Tsiroanomandidy Region (the western fringe of the Central Highlands) between 1984 and 1986, *An. mascarensis* was reported as the second most prevalent *Anopheles* mosquito after *Anopheles maculipalpis.*^35^ Notably, a similar survey covering the entirety of Malagasy Island from January 1982 to May 1988 revealed the presence of *An. mascarensis* in multiple sites across the country, confirming a higher prevalence of this mosquito species in the eastern region of Madagascar, as well as its absence or scarce occurrence in the north and south.^36^ Finally, after the 1980s malaria epidemics in the Central Highlands, a specific survey on malaria vectors was conducted in 1987 in three villages located near Antananarivo.^18^ The abundance of *An. mascarensis* was similar to that of the suspected malaria vectors *An. gambiae* and *An. funestus*, but far lower than that of *Anopheles coustani* (*An. coustani*) and *Anopheles squamosus* (*An. squamosus*), both of which have been recognized as potential secondary malaria vectors in Madagascar.^37^ For the Comoros, *An. mascarensis* larvae were found in Anjouan in 1974,^38^ and adults were found in Mayotte in 1972.^39^ A more recent survey (2008–2012) revealed the continued presence of *An. mascarensis* in Mayotte.^40^

**Figure 4.**
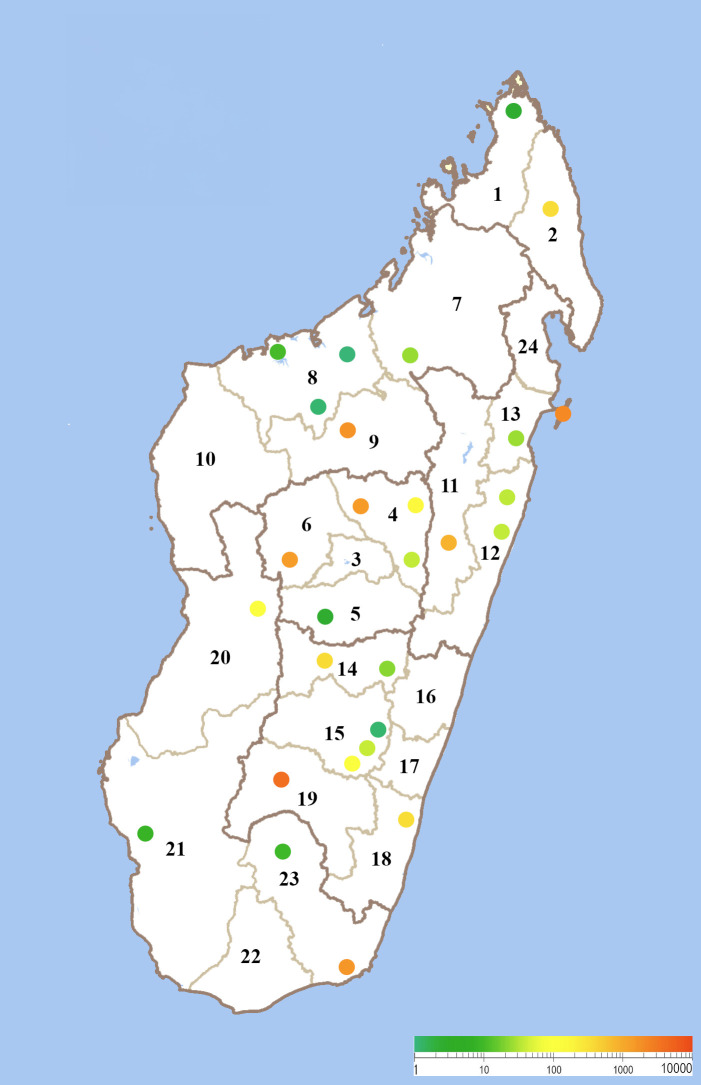
Distribution map of *Anopheles mascarensis* collected from 1989 to 2015. Data were extracted from the mosquito database of the Medical Entomology Unit at the Institut Pasteur de Madagascar. The limits of the 24 different administrative regions are indicated. Colored dots correspond to the 28 sampled districts (Supplemental Table 2), and abundance is indicated according to the colored scale.

To complement this information, records of *An. mascarensis* collated in the mosquito database of the Medical Entomology Unit at the Institut Pasteur de Madagascar were extracted. The samples correspond to entomological surveys performed from 1989 to 2015 using diverse methods, including odor-baited traps (human and animal), CDC light-traps, Muirhead–Thomson pit traps, catches in stable and outdoor animal shelters, human landing catches (HLCs), and indoor pyrethroid spraying. Only a subset of this database has been published.^41^^–^^44^ The entomological surveys were performed in 126 localities (or villages). *Anopheles mascarensis* was found in 93 localities distributed across 28 districts, covering 18 of the 24 administrative regions of Madagascar (Supplemental Table 1). According to these records, 13,026 *An. mascarensis* were caught during these 26 years of entomological surveys (Supplemental Table 2). With the exception of the years 1989, 1993, 1994, and 2005, *An. mascarensis* was caught every year. A plot of the abundance of *An. mascarensis* by district is presented in Figure 4. However, it is important to note that some places were surveyed only once, whereas others benefited from longitudinal surveys. This map confirms the presence of *An. mascarensis* in areas where it was already present in the 1960s. The absence of records in some areas, when compared with the 1960s map, does not necessarily indicate a decline in its distribution over time. Indeed, most surveyed localities were included in malaria surveillance activities over the period spanning from 1989 to 2015.

### Biology.

#### Breeding sites.

*Anopheles mascarensis* usually breeds in renewable watercourses, particularly in transplanted or uncropped rice fields.^8^^,^^45^ The larvae dwell in both forest streams and water bodies in deforested areas. They also breed in irrigation canals and streams.^8^^,^^26^^,^^46^ In Anjouan (Comoros), they were found in watercourses^38^ and in marshy areas, such as in Mayotte.^40^ *Anopheles mascarensis* larvae can tolerate streams in which the current reaches 40 cm per second; however, they are primarily found in still water ponds.^8^ In sunny rice fields, they look for shaded areas provided by shrubs, trees, or any upright vegetation. Regarding association with other malaria vectors, *An. mascarensis* larvae are commonly found alongside *An. gambiae* s.l. and *An. coustani* larvae, but rarely with *An. funestu*s larvae.^8^

#### Trophic and resting behavior.

**Figure 5.**
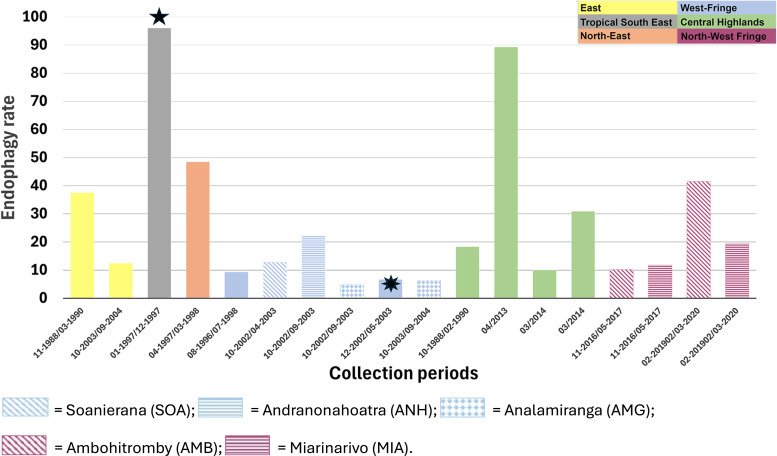
Endophagic rate of *Anopheles mascarensis* in different regions of Madagascar. Within the western and northwestern fringe regions, villages that were sampled during different periods are labeled. At the site with the eight-pointed star, human landing catches were performed, along with captures using Mbita traps, corresponding to the pooled data from three villages (SOA, ANH, and AMG; Supplemental Table 3). Five-pointed star = the weather was highly windy, possibly affecting the outdoor collection; eight-pointed star = the lowest value reported was less than 6%.

In his seminal work, which was conducted in several regions of Madagascar, Grjebine concluded that *An. mascarensis* is a polyphagous and predominantly zoophagic species that feeds on humans when cattle are rare.^8^ He reported that anthropophagy can vary from 0% to 85%, with geographic variations possibly linked to agricultural and cultural practices. The published reports covering the period from 1988 to 2024 (Table 1) provide limited information on the trophic behavior of *An. mascarensis*, based on blood meal analysis. Only three publications revealed data indicating an anthropophagic rate that varies from 0% to 7.9%.^41^^,^^42^^,^^47^ Most mosquitoes feed on bovines. The HLC information from the same set of publications and from the Institut Pasteur de Madagascar database covering the years from 1989 to 2015 confirms Grjebine’s report,^8^ which reveals that *An. mascarensis* is exophagic, preferentially feeding outside (Supplemental Tables 2 and 3, Figure 5), with two exceptions. First, the populations collected in Fort Dauphin were highly endophagic (rates of 87.87–96.04%). As hypothesized by Marrama et al.,^11^ the endophagic behavior of *An. mascarensis* in Fort Dauphin was likely due to constant high winds blowing in that area. The other population with a high endophagic rate (89.29%) was collected in the Central Highlands (Figure 5, Supplemental Table 3). However, the sample size was limited (*n* = 28), and the endophagic rate of mosquitoes from neighboring villages was less than 31% (Figure 5, Supplemental Table 3). Regarding the resting behavior of *An. mascarensis*, both unpublished (Supplemental Table 2) and published data (Supplemental Table 4) confirm that the surveyed populations are preferentially exophilic, in agreement with Grjebine’s report.^8^ The sample size of the single record revealing a high endophilic rate (83.33%) was quite low (*n* = 6), with no additional information on the climatic conditions at the time of the survey (i.e., high wind).^48^ Globally, *An. mascarensis* has a tendency for exophagy (feeding outside) and exophily (resting outside).

### Insecticide resistance.

Possibly because of its exophilic and exophagic behavior, as well as its known zoophily, *An. mascarensis* was not included in systematic insecticide resistance assays until recently. Past records have revealed the high frequency of nulliparous females after insecticide treatments, suggesting that in the 1960s, this mosquito species was fully susceptible to insecticides.^8^ To the best of the authors’ knowledge, a single recent report addressed *An. mascarensis*’ susceptibility status to insecticides.^49^ From indoor-collected adults at four sites in the east and southeast of Madagascar, it can be concluded that in those areas, *An. mascarensis* is fully susceptible to permethrin, deltamethrin, and pirimiphos-methyl, despite small sample sizes (18 < *n* <65). Similar results were obtained in the president malaria initative Africa indoor residual spraying project.^50^

### Vector role of *Anopheles mascarensis*.

#### Malaria.

In Madagascar, it has long been believed that malaria transmission is only due to *An. funestus* and *An. gambiae* sensu lato. Intensive entomological surveys conducted until the 1960s did not reveal any *An. mascarensis* mosquitoes bearing sporozoites in dissected salivary glands, either on the east coast (4,350 mosquitoes) or the west coast (959 mosquitoes).^7^ In contrast, because of their abundance, *An. coustani* and *An. squamosus* were suspected to act as local malaria vectors in areas with residual malaria, where *An. gambiae* and *An. funestus* were barely detected.^37^ On the basis of the abundance criterion, Fontenille et al.^10^ suspected that *An. mascarensis* could be an important malaria vector in the Sainte Marie island, where malaria prevalence reached 85% in children aged 10–15 years old. Thanks to the development of the ELISA technique for detecting *Plasmodium* circum-sporozoite protein in preserved mosquito heads and thoraces,^51^ they showed that *An. mascarensis* was indeed a vector of human *Plasmodium* with the detection of *Plasmodium falciparum* (*P. falciparum*) in 14 mosquitoes among 1,864 tested, corresponding to a mean sporozoitic index (SI%) of 0.75. During that survey, infection of *An. gambiae* s.l. by any of the four human *Plasmodium* species present in Madagascar was high, with 165 infected mosquitoes among 9,453 tested (mean SI% value of 1.78).^10^ In the following years, the systematic detection of *Plasmodium* sporozoites in *An. mascarensis*, as determined via ELISA or polymerase chain reaction (PCR), was performed during entomological surveillance campaigns and malaria epidemiology studies. This led to the identification of evidence suggesting that in the tropical southeast of Madagascar, *An. mascarensis* was the main malaria vector, even if *An. gambiae* s.l. and *An. funestus* were present.^11^ Indeed, *An. mascarensis* was responsible for two-thirds of the infective bites.

A comprehensive summary of the published data from Table 1 and Figure 2 is presented in Table 2, which includes any instances in which the three species, *An. mascarensis*, *An. gambiae s.l.*, and *An. funestus*, were collected at the same site and period via HLC and subsequently tested for *Plasmodium* sporozoites. From these published data and for ease of comparison, an annual entomologic inoculation rate (EIR) was calculated, although transmission does not occur year-round at every site and data were not collected over the same time period. These collated data confirm the important contribution of *An. mascarensis* to malaria transmission in the eastern part of Madagascar, likely due to the abundance of this species. Notably, Le Goff et al.^52^ reported the detection of 11 *P. falciparum*-positive *An. mascarensis* mosquitoes out of a total of 2,463 specimens (SI%: 0.45) in the east and tropical south, according to surveys performed between 1997 and 2003. In contrast, they reported only one *P. falciparum*-positive *An. mascarensis* mosquito out of a total of 2,214 specimens (SI%: 0.045) in the western fringe of Madagascar, over the same time frame. In the Central Highlands and northwestern fringe, no *Plasmodium* sporozoites were detected in *An. mascarensis*; however, both *An. gambiae* s.l. and *An. funestus* were found to be infected with *Plasmodium* (Table 2).^9^^,^^53^

**Table 2:** Summary of published data on the detection of *Plasmodium* sporozoites in *Anopheles mascarensis*, *Anopheles gambiae*, and *Anopheles funestus* collected at the same site during the same period

Regions	Species/Site/Date	ma	SI%	Annual EIR	Number Tested for Plasmodium via ELISA	Plasmodium Species	References
East coast	Sainte Mari**e** (November 1988/March 1990)						
	*An. mascarensis*	18.08	0.75	49.48	1,864	Pf	10
	*An. gambiae*	16.48	1.78	107.4	9,453	Pf, Pv, Pm, Po	
	*An. funestus*	0.47	0.59	1.01	338	Pf	
	Saharevo (October 2003/September 2004)						
	*An. mascarensis*	0.74	0.75	2.03	268	Pf, Pm	42
	*An. gambiae*	0.39	0.47	0.67	211	Pf	
	*An. funestus*	0.65	1.58	3.75	633	Pf, Pv	
Tropical southeast	Esana (January 1997/December 1997)*						
	*An. mascarensis*	7.57	0.89	24.6	677	Pf	11
	*An. gambiae*	2.66	N/A	9	N/A	Pf	
	*An. funestus*	0.66	N/A	3	N/A	Pf	
Northeast	Mandritsara (April 1997/March 1998)						
	*An. mascarensis*	0.83	NT	NT	NT	–	48
	*An. gambiae*	1.34	2.13	10.41	47	Pf	
	*An. funestus*	4.48	1.31	21.42	842	Pf	
Western fringe	Ambohimena (August 1996/July 1998)						
	*An. mascarensis*	0.61	NT	NT	NT	–	
	*An. gambiae*	0.35	0.00	0.00	871	–	
	*An. funestus*	6.73	0.2	4.91	10,753	Pf	
	Soani**e**rana (October 2002/April 2003)						
	*An. mascarensis*	1.04	0.86	3.26	116	Pf	52
	*An. gambiae*	N/A	N/A	N/A	N/A	–	
	*An. funestus*	N/A	N/A	N/A	N/A	–	
	Andranonahoatra (October 2002/September 2003)						
	*An. mascarensis*	0.28	0.00	0.00	54	–	52
	*An. gambiae*	N/A	N/A	N/A	N/A	–	
	*An. funestus*	N/A	N/A	N/A	N/A	–	
	Analamiranga (October 2002/September 2003)^†^						
	*An. mascarensis*	3.17	0.00	0.00	722	–	52
	*An. gambiae*	0.72	0.00	0.00	672	–	
	*An. funestus*	9.15	0.17	5.68	4,056	Pf, Pv	
	Analamiranga (October 2003/September 2004)						
	*An. mascarensis*	1.91	0.00	0.00	590	–	41
	*An. gambiae*	0.59	0.00	0.00	363	–	
	*An. funestus*	7.55	0.07	1.93	2,774	Pf	
Central highlands	Manarintsoa (October 1988/February 1990)						
	*An. mascarensis*	1.14	0.00	0.00	178	–	
	*An. gambiae*	1.67	0.11	0.67	2,759	Pf, Pv, Pm	
	*An. funestus*	0.09	0.47	0.15	214	Pf	
	Ankazobé (December 1989/April 1990)^‡^						
	*An. mascarensis*	N/A	0.00	0.00	63	–	9,12
	*An. gambiae*	0.96	0.87	3.05	115	Pf, Pv, Pm	
	*An. funestus*	20.82	0.92	70.08	2,498	Pf, Pv, Pm	
Northwestern fringe	Andriba (December 1989/April 1990)^‡^						
	*An. mascarensis*	N/A	0.00	0.00	180	–	9,12
	*An. gambiae*	8.47	0.16	0.13	611	Pf, Pv	
	*An. funestus*	8.83	1.8	58.01	276	Pf, Pv	
	Andriba (November 2016/April 2017)^§^						
	*An. mascarensis*	0.92	0.00	0.00	59	–	53
	*An. gambiae*	5.81	2.41	51.11	374	Pf, Pv	
	*An. funestus*	2.53	1.42	13.11	212	Pf, Pv	

The colors used in the table match the colors used in Figures 2 and 5, as well as the colors used in the Supplemental Tables.

EIR = entomologic inoculation rate (EIR = ma*SI%/100); ma = bite/man/day, including both indoor and outdoor biting; N/A = not available; NT = not tested; Pf = *Plasmodium falciparum*; Pm = *Plasmodium malariae*; Po = *Plasmodium ovale*; Pv = *Plasmodium vivax*; SI% = sporozoitic index. See Supplemental Table 6 for more detailed data.

* Sporozoitic index data for *An. gambiae* and *An. funestus* were extracted from the publication text.^11^

^†^ Infection data for *An. gambiae* and *An. funestus* were extracted from Robert et al.^41^

^‡^ Data were extracted from both Fontenille and Fontenille and Campbell.^9^^,^^12^

^§^ *Plasmodium* detection was performed using real-time polymerase chain reaction testing.

Whether abundance, vectorial competence, or both contribute to the role of *An. mascarensis* as a malaria vector is an unanswered question. To address the contribution of abundance, the proportion of *An. mascarensis* was compared among the three major Malagasy vectors, *An. mascarensis*, *An. gambiae* s.l., and *An. funestus*, across different investigated regions in Madagascar. To accomplish this aim, mosquito numbers captured by HLC were only extracted from published records. The relative abundance of *An. mascarensis* varies greatly geographically (Supplemental Figure 1, Supplemental Table 5). It is predominant in the tropical southeast, representing roughly 50% of malaria vectors in the east and Central Highlands. Therefore, in those regions, its high abundance is likely responsible for its role as a malaria vector. In all other regions where its relative abundance is low, *An. mascarensis* was not found to bear *Plasmodium* sporozoites, except in Soanierana. In this locality, despite its low abundance, *An. mascarensi*s co-occurring with *An. gambiae* s.l. can maintain a weak malaria transmission in the western fringe region in the absence of the main vector, *An. funestus.*^52^ In conclusion, the currently available dataset suggests that abundance is the main driver of malaria transmission by *An. mascarensis.*

### Filariasis.

Although Grjebine mentioned that *An. mascarensis* was found with metacyclic microfilariae on the east coast,^8^ likely caused by *Wuchereria bancrofti* (*W. bancrofti*), this information could not be traced to an accessible publication. However, Brunhes and collaborators^54^^,^^55^ were able to experimentally infect two female *An. mascarensis* mosquitoes fed on the blood of a patient infected with *W. bancrofti.*

### Arboviruses.

In a systematic search and inventory for arboviruses circulating in Madagascar, *An. mascarensis* was found to be naturally infected by the Ngari virus in the Anjiro Region, which is located 72 km east of Antananarivo, on two occasions.^36^ Originally isolated from Senegalese *Aedes simpsoni* males in 1979, the Ngari virus (Bunyaviridae family, Orthobunyavirus genus) was responsible for hemorrhagic fever outbreaks in humans, from whom it was first isolated in 1993.^56^ Until recently, *An. mascarensis* was not implicated as a putative vector for other arboviruses. However, in 2021, after an outbreak of Rift Valley fever virus (RVFV) on the east coast of Madagascar, high numbers of captured *An. mascarensis* were found to be harboring RVFV RNA via PCR testing in 9 of 121 pools of roughly 10 females each.^57^ Determining the actual role of *An. mascarensis* in RVFV transmission requires additional investigation through virus isolation from wild mosquitoes and experimental infections in the laboratory.

### *Anopheles mascarensis*: a potential complex of sibling species.

In his careful work, in which he aimed to demonstrate that *An. mascarensis* is sometimes confused with *An. marshalli*, Chauvet established biometric diagnosis parameters.^3^ These biometric characteristics led him to demonstrate that populations of *An. mascarensis* from the east coast (Ivoloina) differ from those collected near Antananarivo in the Central Highlands. These characteristics included the color of the palps (light versus dark), wing length, and banding pattern. Already at that time (1962), indicating these observed geographical variations, Chauvet wrote that *An. mascarensis* was possibly a species in full evolution. Based on the seminal work of M. Coluzzi on the *An. gambiae* s.l. mosquito, Chauvet’s observations were indeed suggestive that *An. mascarensis* could be a complex of species. Later on, Fontenille and Campbell^9^ provided additional behavioral evidence that *An. mascarensis* populations from the east coast differed from those in the Central Highlands: one population is anthropophilic, whereas the other is zoophilic. They suggested that *An. mascarensis* is a complex of sibling species with different vectorial capacities. Furthermore, Le Goff et al.^52^ reported considerable differences between the vector role and biology of *An. mascarensis* populations from the west and those from the east coast. However, a study on the genetic polymorphism of *An. mascarensis* from Madagascar, conducted using random amplified polymorphic DNA PCR testing, revealed that there was no genetic differentiation between the populations from the coast and those from the Central Highlands.^58^ With access to novel technologies such as next generation sequencing, it would be wise to reassess the genetic makeup of different populations of *An. mascarensis* to more precisely document the possible existence of sibling species within the *An. mascarensis* species.

### Miscellaneous information.

In this section, three sets of published data not discussed in the previous sections are reported. First, an odor-baited entry trap investigation conducted in three villages located in the western, eastern, and southern fringes of Madagascar revealed that *An. mascarensis* is preferentially attracted to calves.^59^ Secondly, Zohdy and collaborators conducted a study in six villages located near the Ranomafana National Park in the southeastern humid tropical forest, with the aim of testing the efficacy of a human microbiota-derived attractant in CDC light traps to capture *Anopheles* mosquitoes. The comparative assay (with or without the attractant) revealed that the attractant has no effect on *Anopheles* trapping, including *An. mascarensis*. Their study indicates that more *An. mascarensis* were trapped in agricultural sites rather than in villages or forest sites.^60^^,^^61^ Surprisingly, in another study performed in the same region 3 years later using CDC light traps, no *An. mascarensis* were captured.^62^ Thirdly, an innovative strategy was developed by Tedrow et al.,^63^ who combined a modified barrier screen for outdoor *Anopheles* capture and a multiplex molecular assay capable of identifying mosquito species, blood meal origin, and *Plasmodium* sporozoites. In their study conducted in the Tsiroanomandidy District in December 2017 and April 2018, an equal proportion of *An. mascarensis* had fed on humans in December and in April (35%), whereas the proportion that had fed on cows shifted from 35% in December to 50% in April. Two individual An. *mascarensis* mosquitoes were found to be infected with *P. falciparum* and both *P. falciparum* and *Plasmodium vivax*, respectively.

## CONCLUSION

The data analyzed in this review support the theory that *An. mascarensis* is a complex of sibling species, given its abundance relative to *An. gambiae* s.l. and *An. funestus*, which share similar biotopes, endophagic behavior, and vectorial capacity (estimated as annual EIR). Supporting the observations and data reported by both Chauvet and Fontenille and Campbell, the collated data highlight a notable difference between populations from the east coast and those from the Central Highlands of Madagascar. Testing the vectorial competence of each population against a reference strain of *P. falciparum* could provide an additional phenotypic tool. With advances in molecular tools, testing the hypothesis that *An. mascarensis* is a complex of sibling species is clearly within reach. For instance, using a set of nuclear and mitochondrial markers, such as 18S-internal transcribed spacer and cytochrome oxidase subunit I, could aid in further classification. The recent publication of the first genome of *An. mascarensis* will undoubtedly provide additional information and tools (April 2025; https://www.ncbi.nlm.nih.gov/bioproject/1197942). Furthermore, it would also be interesting to test this hypothesis with *An. mascarensis* populations from the Comoros Archipelago.

## Supplemental Materials

10.4269/ajtmh.24-0697 Supplemental Materials
