# Supplementary material for: Anopheles mascarensis, a Malaria Vector Endemic to Madagascar and the Comoros Archipelago: A Review
Source: Am J Trop Med Hyg. 2025 Oct 28;114(2 Suppl):42–51. doi: 10.4269/ajtmh.24-0697 (PMC12874975; doi:10.4269/ajtmh.24-0697)

Supplemental Figure 1. ***An. mascarensis*** **relative abundance varies greatly geographically, as shown in this figure.**

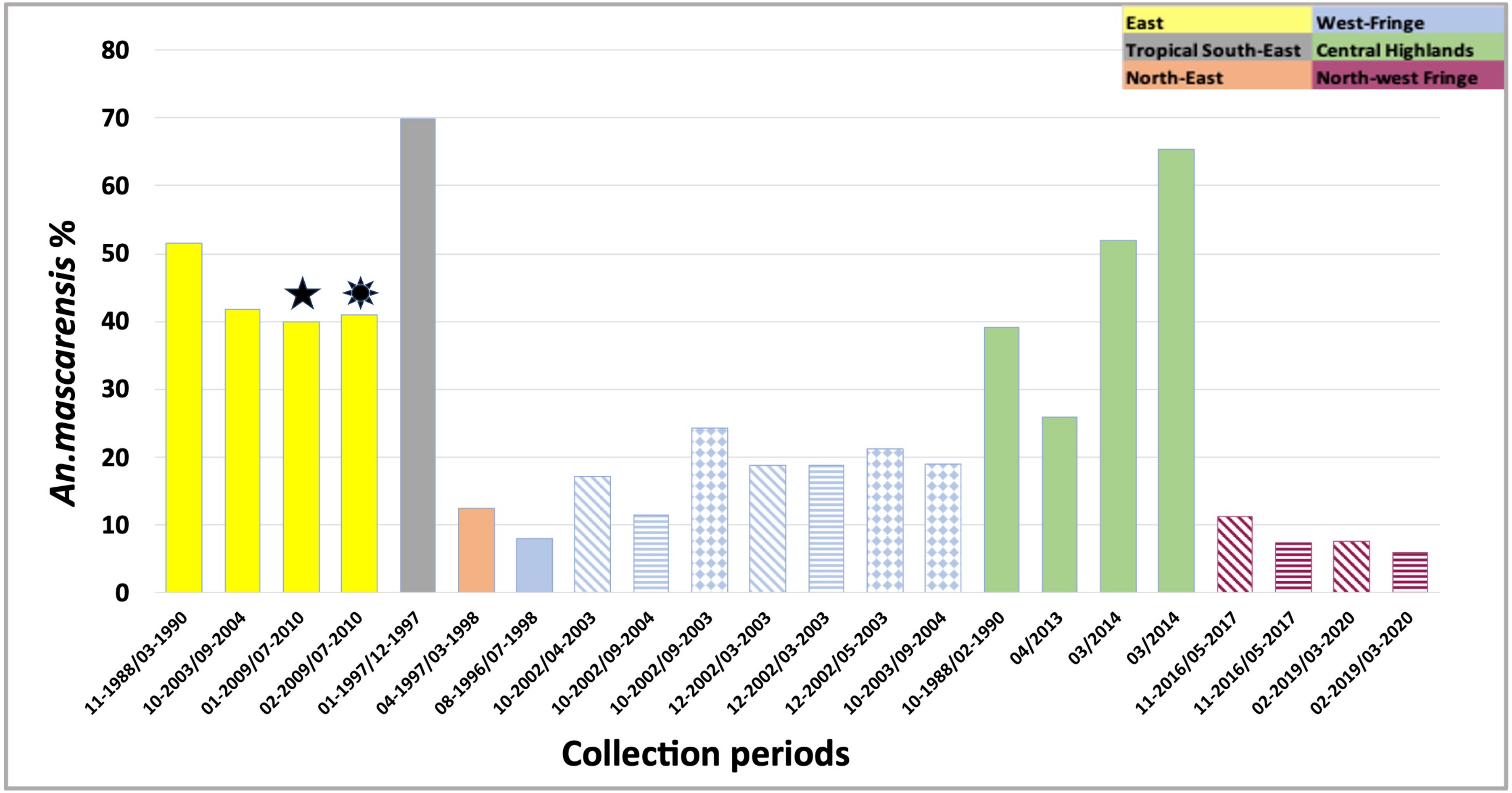

Supplement: Supplemental Materials [file tpmd240697.SD2.pdf]
